# ‘We feel abandoned out here’: teamwork dilemmas among rural health professionals in distributed emergency settings

**DOI:** 10.1186/s13049-026-01639-9

**Published:** 2026-06-18

**Authors:** Hanna Morian, Magnus Hultin, Johan Creutzfeldt, Hanna Dubois, Maria Härgestam

**Affiliations:** 1 Department of Nursing, Umeå University, Umeå, Sweden; 2 The Swedish Centre for Rural Health, Region Västerbotten, Storuman, Sweden; 3 Department of Diagnostics and Intervention, Anaesthesia and Critical Care Medicine, Umeå University, Umeå, Sweden; 4 Centre for Advanced Medical Simulation and Training, Karolinska University Hospital, Stockholm, Sweden; 5 Department of Clinical Science, Intervention and Technology, Karolinska Institutet, Stockholm, Sweden; 6 Department of Clinical Science and Education, Karolinska Institutet, Södersjukhuset, Stockholm, Sweden

**Keywords:** Distributed teams, Telemedicine, Emergencies, Teamwork, Leadership, Communication, Collaboration, Focus groups, Interviews, Discourse psychology

## Abstract

**Background:**

In rural northern Sweden, community hospitals serve as essential first-response centres. During emergencies, general practitioners remotely collaborate on-call with on-site nurses via videoconferencing. This setup of geographically distributed teams enables healthcare delivery across long distances. However, little is known about how healthcare professionals make sense of and negotiate roles and responsibilities in such distributed emergency settings. Hence, the aim of this study was to analyse how healthcare professionals position themselves and others in interprofessional teams when collaborating in distributed emergency settings in rural areas.

**Methods:**

Interprofessional focus group interviews (*n* = 17) were conducted with staff (one nursing assistant, one registered nurse and one physician per focus group) at community hospitals in rural northern Sweden, following full-scale, simulated, in-situ team training. The analysis was inspired by Billig’s concept of ideological dilemmas and Wetherell’s concept of interpretative repertoires.

**Results:**

Participants drew on three interpretative repertoires—Involvement, Responsibility and competence, and Control and dependency—to account for teamwork in distributed emergency settings. Across these repertoires, participants accounted for involvement as both enabled and difficult to sustain, responsibility as not readily transferred, and control as limited and dependent on others’ accounts. These ways of accounting reflected ongoing dilemmas, as professionals positioned themselves in relation to competing demands when acting under conditions of distance, uncertainty and mediated access to the clinical situation.

**Conclusions:**

Distributed teamwork reshapes the conditions for collaboration in emergency care, as roles and responsibilities become continuously negotiated in relation to tensions concerning involvement, responsibility and control. As a result, collaboration is unevenly achieved and cannot be taken for granted.

**Clinical trial number:**

Not applicable.

## Background

Effective teamwork is crucial in delivering high-quality healthcare, as communication errors and poor teamwork often lead to adverse outcomes [1]. In an emergency, strong leadership that promotes shared mental models, clear communication and defined roles is essential to ensure precise coordination around the patient [2, 3]. While effective teamwork is vital for patient safety [2, 4, 5], team dynamics are often described under the assumption that all team members, including the team leader, are physically present in the same location as the patient [2]. This perspective may not fully capture the dynamics of distributed teams, in which team members are geographically distributed and use technology to communicate [6]. In a distributed team, team communication must adapt to the physical absence of team members and the use of technology [7], while leadership, roles and responsibilities may need to be redefined and negotiated across distance.

Research on teamwork in distributed teams has focused on ad-hoc student teams dealing with hypothetical tasks [8] and settings outside healthcare [9], often with inconsistent findings [10–13]. Although distributed teams face challenges similar to those of co-located teams, their limited face-to-face interaction [7] can complicate teamwork [14] and increase workload [15]. Furthermore, communication and coordination can be more challenging without established trust or familiarity, which are difficult to build in distributed teams [16]. Advanced research on distributed teams has been conducted in fields such as aviation [17] and business [18], but healthcare’s unique demands, which include urgent tasks and patient safety, require empirical studies specific to this context. Moreover, previous research in non-healthcare settings has primarily focused on the structural and functional aspects of teamwork, such as communication, coordination and workload [19, 20], while paying less attention to how team members make sense of and negotiate their roles in distributed settings.

In rural northern Sweden, the absence of conventional hospitals, combined with challenges that include long distances, limited accessibility and severe weather conditions, has driven the need for innovative healthcare solutions [21]. As one such solution, community hospitals (CHs) serve as first-response centres in sparsely populated areas [22]. Despite their limited capacity, CHs operate 24/7, offering various services that include primary care, emergency treatment and diagnostic capabilities [22]. During on-call periods, CHs are supported by distributed teams [6], with general practitioners (GPs) participating remotely while nursing assistants (NAs) and registered nurses (RNs) provide on-site care. Thus, the team members in these distributed teams collaborate through technology [21], which enables GPs to manage multiple CHs simultaneously and remotely, addressing emergencies across long distances. This creates a context in which roles and responsibilities are not always clear and must be worked out in practice by professionals collaborating at a distance, through technology and across different professions.

Although distributed teams may face unique challenges [15], there is limited understanding of how healthcare professionals make sense of their roles in practice in rural emergency settings. Such processes are central in distributed emergency care, where roles are not always clearly defined but must be actively worked out in interaction [19]. To address this limitation, a discourse psychology (DP) approach was used to explore how teamwork is constructed in distributed emergency settings in rural areas. Rather than focusing on what teamwork is, DP makes it possible to examine how teamwork is made meaningful by participants [23]. In this study, we analyse how healthcare professionals talk about teamwork in distributed emergency settings, focusing on how they account for, justify and negotiate roles and responsibilities. The analysis focuses on *interpretative repertoires* [24], which are understood here as recurring ways of talking about teamwork; on *subject positions* [24], which reflect how roles and responsibilities are constructed; and on *ideological dilemmas* [25], which capture tensions between competing expectations in teamwork within this context.

The aim of this study was therefore to analyse how healthcare professionals position themselves and others in interprofessional teams when collaborating in distributed emergency settings in rural areas.

## Methods

### Design

This study is part of a broader research programme titled Teamwork in Geographically Dispersed Emergency Teams in Rural Settings (TIGER), which aims to improve rural emergency care by redefining teamwork in geographically dispersed teams [26–32]. The aim of this study was to analyse how team members position themselves and others within interprofessional teams while collaborating in a distributed setting during simulated emergencies. We employed a qualitative design inspired by DP [32]. To support completeness in reporting, we used the Consolidated Criteria for Reporting Qualitative Results (COREQ) guidelines [33].

### Setting

This study was conducted in the rural inland areas of northern Sweden and involved seven CHs in southern Lapland. These CHs are essential for providing first-response care, being situated in one of Europe’s least densely populated regions, with less than one person per square kilometre in some areas [21]. Moreover, the rural and often elderly population presents unique healthcare demands. Staff members at CHs manage a range of chronic and acute conditions [34] – a challenge compounded by a shortage of healthcare workers [35]. Some CHs are up to 350 km away from the nearest urban hospital with specialised care [22]. Ambulance services are based near CHs, yet residents often face long waiting times due to the long distances, which are made worse by challenging weather conditions such as snowy roads. With no private healthcare alternatives, the CHs serve nearly all local residents (500–6000 per rural community). These CHs are primary healthcare centres, providing family medicine, maternal care, minor surgery, palliative care and 24/7 emergency care.

Staff at CHs include NAs, RNs, resident physicians, GPs, midwives, physiotherapists and occupational therapists [22]. These professions differ in educational background and scope of practice. In Sweden, NAs complete vocational training at the secondary school level [36], while RNs hold at least a bachelor’s degree after 3 years of university studies. Many RNs pursue additional specialist training, such as in district nursing or midwifery, which requires an additional 1.5–2 years to achieve a master’s degree [37]. GPs complete a 5.5-year master’s degree, followed by a 2-year internship and 5 years of specialist training [38].

RNs and NAs provide immediate care on-site during on-call hours, while GPs are on call for a larger area and connect via videoconferencing to join the distributed team. If videoconferencing cannot be utilised, telephone consultation may be the only option, as travelling between CHs for medical assessments is often too time-consuming in acute situations, risking delays that could prevent timely intervention [21].

### Data collection

#### Participants

All NAs, RNs and GPs employed at all seven rural CHs in northern Sweden were eligible for inclusion in this study. Recruitment was facilitated through the manager of each CH, who distributed information about the study to all staff. Participation was voluntary and based on staff availability. A total of 51 medical staff members participated: 13 NAs, 21 RNs and 17 GPs. Consequently, 17 three-person focus groups were organised. Of these, 13 focus groups included one NA, one RN and one GP, while the remaining four comprised two RNs and one GP. No participants declined or dropped out. Participants’ ages ranged from 35 to 51 years (median 42), with most being female (*n* = 42/51). Participants’ work experience spanned 6–26 years (median 16), and 26% had prior experience working in a distributed setting. Detailed participant characteristics are presented in Table 1.

**Table 1:** Participant characteristics

	All participants *n* = 51	Nursing assistants *n* = 13	Registered nurses *n* = 21	General practitioners *n* = 17
Age, median (Q1–Q3)Age, mean (SD)Not disclosed *n* (%)	42 (35–51) 43 (12) 1 (2)	37 (28–49) 39 (13)	38 (32–54) 41 (13)	43 (40–55) 47 (9) 1 (6)
Female *n* (%)	42 (82)	12 (92)	19 (90)	11 (65)
Male *n* (%)	9 (18)	1 (8)	2 (10)	6 (35)
Experience in healthcare				
Year median (Q1–Q3) Not disclosed *n* (%)	16 (6–26) 2 (4)	20 (5–26) 1 (8)	16 (5–27)	17 (10–27) 1 (6)
No previous experience in simulation training, *n* (%) Previous experience in simulation training < 10 events, *n* (%) Previous experience in simulation training ≥ 10 events, *n* (%) Not disclosed *n* (%) Previous experience working in a distributed team, *n* (%) Not disclosed *n* (%)	7 (14) 27 (53) 1 (2) 16 (31) 14 (29) 3 (6)	2 (33) 4 (67) 7 (54) 2 (15)	4 (25) 13 (76) 4 (19) 7 (35) 1 (5)	1 (8) 10 (84) 1 (8) 5 (29) 5 (33) 2 (12)

#### Pre-interview simulated team training

**Fig. 1:**
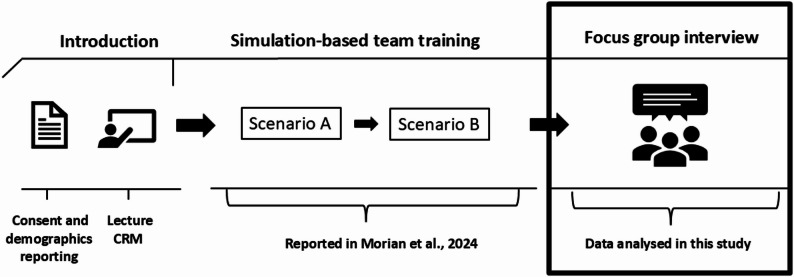
Data collection, including simulation-based team training prior to interviews. The half-day session included an introduction, simulation-based team training and focus group interviews. The simulation-based team training consisted of two scenarios (**A** and **B**) and provided a shared context for the subsequent interviews. Details of the simulation design and procedures are reported elsewhere [27]. Focus group interviews were conducted immediately after the team training and are analysed in the present study

Data were collected between 2019 and 2021 as part of the TIGER programme. During this period, each team participated in a single half-day session that included both simulation-based team training [27] and a subsequent focus group interview conducted immediately after the training (Fig. 1). Participants remained within the same team constellation during their team training and focus group interview.

Prior to the simulation, participants received a brief introduction to teamwork principles, including crew resource management [39]. Each team then participated in two 20-minute scripted scenarios involving a standardised patient (a trained individual acting as a patient) [40] with deteriorating vital signs at their CH. The scenarios comprised two emergency cases: urosepsis (Scenario A) and myocardial infarction (Scenario B). Scenario A was conducted in a co-located team setup, whereas Scenario B involved a distributed team setup. During the distributed scenario, RNs and NAs were physically present with the patient, while the GP participated remotely via videoconferencing. Further details and results are reported elsewhere [27].

In the present study, only data from the focus group interviews are analysed. The simulation is described here, as it preceded the interviews and provided a shared reference point – particularly since not all participants had prior practical experience of using the distributed model in emergency situations. During the interviews, participants drew on both the scenarios and their everyday clinical experiences.

#### Focus group interviews

Between 2019 and 2021, we conducted focus group interviews at seven CHs. A focus group format was chosen to encourage discussions and gather diverse perspectives across various professions [41]. The interviews took place in designated rooms separated from the CH’s regular activities to minimise disturbance at each CH. Participants were asked to discuss their experiences working and collaborating in an interprofessional team when managing a patient in an emergency within a distributed team setting. Although the interview questions were informed by the preceding simulation-based team training, participants were encouraged to draw on both their everyday work experiences and the training scenarios. In line with the DP approach, the analysis focused on how participants constructed teamwork in talk, regardless of whether their accounts were grounded in the simulation or everyday practice.

A semi-structured interview guide with open-ended questions was used, including the following questions: ‘How did you perceive the collaboration in the distributed setting?’ ‘Can you tell me how you experienced communication in the distributed setting?’ ‘How did you view your role in the team in the distributed setting?’ and ‘How did you perceive leadership in the distributed setting?’. Follow-up questions such as ‘Could you please elaborate on this?’ were employed to help the participants deepen and expand their narratives. The interviews were audio-recorded and typically lasted around 60 min. In all interviews, two researchers attended: HD and MHä or HM. One of the authors (MHä or HM) conducted the interviews, while the other (MHä or HD) assumed the role of observer and note-taker. Verbatim transcriptions were created from audio recordings of the interviews.

### Analysis

In this study, DP was employed to analyse the transcripts. DP can be used to investigate how language and interactions both shape and are shaped. In DP, language is viewed as a tool for understanding diverse interpretations of the world [23] and as a means of constructing social concepts such as identities and roles, which are seen as dynamic rather than fixed [42]. MHä and HM led the analysis, with regular discussions among all authors to refine the findings. The interviews were conducted in Swedish, and the excerpts were translated into English by the first author.

Overall, the analytical process followed an abductive approach, combining an inductive exploration of the empirical material with the deductive use of DP concepts and moving iteratively between data and theory [43]. Rather than following a linear sequence of steps, the analysis involved continuous movement between parts and wholes, shifting between in-depth engagement with six selected transcripts and a focus on the full dataset of 17 transcripts.

HM reviewed all the transcripts while listening to the recordings to reduce the risk of misinterpretation during analysis. Thereafter, MHä and HM read all the transcripts to gain an overall understanding of the content. To allow close engagement with the data, six transcripts were purposefully selected through discussion between HM and MHä. This selection was based on the variation in how participants talked about distributed teamwork across the dataset, while also considering variation in the participants’ age, gender, ethnicity and experience with distributed settings. The six focus groups in these six transcripts included 18 participants (9 RNs, 6 GPs and 3 NAs), with a predominance of women (*n* = 14). Participants varied in age (23–69 years) and professional experience (1–36 years). Nine participants had prior experience working in distributed settings. The six transcripts were not treated as a separate dataset but as an entry point for a more detailed, iterative analysis of how teamwork was constructed in talk, before returning to the full dataset.

We identified recurring ways of talking about teamwork in distributed settings, with a focus on how the participants used language to account for, justify and make sense of their actions and responsibilities. These discursive patterns were developed into interpretative repertoires, understood as relatively coherent ways of talking about a phenomenon [24]. As emphasised by Wetherell, interpretative repertoires are not fixed or internally consistent; rather, they may be flexible and contradictory and can shift depending on the context in which they are used [24]. The development of these repertoires involved constant comparison within and across interviews.

The analysis resulted in three interpretative repertoires: *involvement*, where the participants talk about distributed teamwork in ways that make participation visible, negotiated and sometimes difficult to sustain; *responsibility and competence*, where teamwork is treated as redistributed, positioning responsibility as both necessary and potentially exceeding established roles; and *control and dependency*, where teamwork is constructed as being shaped by limited access to the clinical context and a reliance on technology and organisational arrangements.

As the analysis progressed, our attention shifted to subject positions, which could involve either preferable (untroubled) or challenging (troubled) role expectations. *Troubled positions* arise when conflicting identities, ambiguous roles or uncertainties about one’s place in a social context occur, whereas *untroubled positions* reflect stable identities and roles that align with context and societal norms [24].

To make sense of the tensions expressed in the participants’ talk, we applied the concept of ideological dilemmas, which reflect tensions between conflicting values, norms or ideas in everyday life. Billig developed this concept, which is useful for capturing ambivalence and contradictions in how people talk about competing expectations and social norms [25]. Identifying dilemmas helped us find moments when such tensions became evident in the participants’ discussions.

All co-authors reviewed and discussed the developing analysis throughout the process. In addition, HD, who attended all interviews, contributed to ensuring that the analysis remained closely aligned with the empirical material.

## Findings

The analysis showed that participants drew on three interpretive repertoires – involvement, responsibility and competence, and control and dependency – as discursive resources to construct their accounts of teamwork by negotiating degrees of involvement, attributing responsibility and competence, and managing relations of control and dependency within a distributed setting. More specifically, through their discourse, participants framed distributed teamwork as enabling participation and support; however, their talk also constructed involvement as unevenly sustained across distance – a formulation that allowed them to account for variations in participation while managing potential accountability. Both formulations draw on the interpretative repertoire of *involvement.* Participants’ talk presented clinical work as redistributed, a discursive strategy that accounted for difficulties in transferring responsibility and extensions of responsibility beyond established roles by attributing these to situational constraints. This strategy draws on the interpretive repertoire of *responsibility and competence*. Finally, the participants constructed their teamwork as being strongly shaped by reduced access to a clinical context and increased reliance on technology and organisational arrangements. Through this rhetorical practice, they were able to justify reduced control while maintaining responsibility for patient care. This practice draws on the interpretive repertoire of *control and dependency*.

To illustrate and provide examples of the subject positions (i.e. troubled or untroubled) taken by the professionals in these discussions, we include excerpts from the transcripts. After each excerpt, the focus group is denoted as ‘FG A–Q’, and the participant’s professional role is marked as ‘NA’, ‘RN’ or ‘GP’. Since some focus groups included two RNs, these are designated as ‘1’ or ‘2’ accordingly. In the excerpts, GPs speak from the distributed team member position, whereas RNs and NAs speak from their on-site position with the patient.

### Involvement

Team members draw on the interpretative repertoire of *involvement* when talking about distributed teamwork as enabling participation and support within the team. One RN positioned the GP’s presence via videoconference during the emergency as supportive and as involving others:

I was surprised by how good it felt to have her [the GP] on the screen. The difference could be, I mean, what you’re used to is that the doctor might be sitting in another room, not actually in the [emergency] room. However, here you [referring to the GP] are present and can see, and you have the computer and can, you know, sit and read, hear everything being said, and so on. (FG N, RN2)

In this account, the RN describes the GP’s presence on the screen as more continuous and accessible than that in a co-located setup, despite the physical distance. The comment that it was ‘surprisingly good’ further suggests that this form of presence is not taken for granted. This construction situates the RN as supported, promoting an untroubled position.

The GPs’ talk forms the distributed setup in similar terms, contrasting it with a co-located setup and emphasising that it allows them to remain actively involved throughout the patient interaction. In these accounts, being ‘involved’ is tied not to hands-on care but to maintaining an overview of the situation, enabling the GP to step back, focus on leadership, and adopt a less stressful position. One GP talked about this as follows:

When you take a step or two back, you get a much clearer view of everything happening in the room, which you might miss if you are right in the middle, especially with all the stress. I do not have that stress because I am sitting far away [in a remote location, connected via video], in a calm environment, with a cup of hot chocolate next to me, staying calm and seeing what needs to be done and what is happening. (FG B, GP)

In this way, distance is associated with enabling clarity and control rather than absence. This formulation allows the GP to take an untroubled position focused on overviewing and decision-making.

The RNs’ talk produces the distributed setting as involving additional responsibilities, which are oriented in different ways. In some accounts, the setting is framed as an opportunity for greater involvement and contribution that allows RNs to take on tasks not typically possible in co-located setups, making it possible for them to adopt an untroubled position. One RN formulates this as follows:

We are so used to not having a physician here during on-call hours. I have built a sense of security through experience, so I am not nervous. I don’t think I work worse when I don’t have a physician present; I still do the same things in many ways. Sometimes, I find it stimulating to have the physician at a distance so that I can do more things. (FG D, RN1)

At the same time, this construction may downplay the extent to which these additional tasks involve taking on responsibilities beyond established roles. The GPs’ accounts draw on the same repertoire, but construct involvement from a different position. As the GPs cannot perform hands-on tasks in the distributed setting, their involvement is framed as requiring delegation to the RNs and a greater focus on communication. One GP put it in this way:

I try to make it clear that this is my responsibility. Just tell me what you hear. You [referring to the RNs] should not feel you have to prove anything – it is my responsibility if I ask someone else to listen and tell me roughly what they hear. If something goes wrong, it is okay, because I am responsible for the assessment and the delegation. So, for me, it feels calm. There is nothing for me to lose. (FG N, GP)

Here, the GP’s language places explicit responsibility on the GP while positioning the RNs as trusted contributors. This framing makes it acceptable for the GP to have trusted contributors – the RNs – carry out delegated tasks the GP would otherwise do, such as listening to the lungs, thereby positioning the RNs as active participants in the assessment rather than in a peripheral role. At the same time, the GP retains overall responsibility for the assessment. This allows the GP to adopt an untroubled position. The NAs’ talk, in turn, frames the distributed setup as enabling them to take on a more direct role in patient care. One NA said,

It’s like I take a bit of a step back when the doctor is in the room because then you [nodding to the GP] are usually by the patient. However, when we were doing telemedicine, it felt like I was closer to the patient. (FG G, NA)

This framing positions the NA as untroubled and even benefitting from the distributed setting, as being physically closer to the patient is presented as a form of involvement that is less available in co-located settings.

Nevertheless, a discrepancy was observed between how collaboration was talked about as taking place on ‘equal terms’ and how the professionals’ participation unfolded in the interviews. While GPs and RNs actively contributed their perspectives, NAs were less vocal in the interviews, and their accounts often aligned with those from other professions. This discrepancy between how the professionals’ discourse constructed their teamwork and their interactions during the interviews sets up a dilemma in which teamwork is framed as inclusive, but not all voices are equally present in the discussions. In this sense, involvement in the distributed clinical setting is treated as dependent on being seen, heard and recognised within the interaction. The same discrepancy was evident in how the other team members oriented to difficulties in participating in this interaction. One GP talked about not knowing whether or not they were part of an ongoing interaction during distributed teamwork:

I didn’t know if they were listening to what I was saying. Did they understand what I just said, and who is now talking to whom? I felt a bit left out, and it doesn’t have to be like that, really. It doesn’t have to be that one feels left out. (FG B, GP)

The same GP further drew on an example of receiving reports from professionals with ‘their backs turned’, pointing to how the on-site RN is oriented towards the patient, rather than the screen, during care. This phrasing constructs a dilemma within the repertoire in which the RN’s focus on the patient positions the GP as peripheral to the interaction. With this dilemma, the GP’s involvement becomes difficult to sustain at a distance, which positions the GP as troubled. A similar dilemma was evident in the RNs’ accounts as well, in which the RNs’ involvement in the interaction was described as fragile and easily disrupted:

The collaboration suffers because I constantly had to run and tell everything to you [referring to the GP] … Then you [referring to RN2] and I lost a bit … I felt entirely left out there. (FG H, RN1)

Here, the RN’s talk presents the involvement as difficult to sustain, since the RN must constantly move between the patient and the GP, placing the RN in a troubled position. In an NA account, bodily positioning is similarly emphasised as shaping involvement in the interaction:

Body language is prevalent in everyday life. You talk just as much with your body as your mouth and eyes. Turning away in some way becomes unsettling. (FG E, NA)

Here, the NA’s phrase ‘turning away’ frames the professionals’ involvement as being disrupted in both directions during an interaction, as a focus on the patient and attention to the GP are presented as equally important in sustaining involvement.

Taken together, these accounts show how the professionals’ talk positions involvement as both central to teamwork and difficult to sustain across distance. The language used positions different team members as outside the group interaction at different moments, such that involvement is constructed as unevenly distributed rather than equally shared.

### Responsibility and competence

The professionals draw on the interpretative repertoire of *responsibility and competence* across distance when framing the physical absence of a GP in the emergency room as creating a gap in both competence and responsibility. The RNs’ discourse positions themselves as the most clinically trained professionals on-site, while referring to hands-on clinical assessment as something that belongs to the GP. Through this way of talking, they frame taking responsibility for clinical decisions on behalf of the patient as both necessary and potentially exceeding the RN role. As a result, responsibility could not be readily handed over from the RNs to the GP in the distributed setting. This framing positions the RNs as exposed and troubled. As one RN put it,

My experience is that I have more responsibility when I am the present nurse [and the GP is remote]. Suppose there is a physician in the room. In that case, I feel a bit more like … it is not ultimately me [who has the responsibility] … even though we are a team, I can sometimes feel that, OK, … there is someone here with higher [medical] competence who maybe can step in and have the competence to do something that I cannot do. Or should not do. (FG Q, RN1)

When drawing on the involvement repertoire, the professionals’ talk constructs responsibility as contained and unproblematic. In contrast, when drawing on the responsibility and competence repertoire, responsibility is oriented as more difficult to manage and less easily transferred. The possibility of handing over responsibility from the RN to the GP if necessary, is framed as central, and the absence of this possibility is described as making responsibility more visible and consequential, even though the GP is digitally present.

The RNs constructed the distributed setting as one in which they managed their typical tasks while also taking on additional tasks usually handled by GPs, such as conducting clinical examinations (e.g., palpating the abdomen or listening to the lungs). At the same time, the RNs talk positioned them as responsible not only for performing clinical assessments but also for conveying them to the remote GP, while simultaneously coordinating the on-site team and maintaining interaction with the patient. In this way, the RNs framed themselves as managing multiple parallel demands: their own clinical tasks, delegated responsibilities from the GP, and ongoing communication with the patient. In one account, an RN oriented to the difficulty of managing these tasks simultaneously:

I felt terrible for the patient because I’m running back and forth like a damn yo-yo … I couldn’t get any flow in my work … I was the one who almost had to run back and forth here to ask questions so that she [the patient] could hear and so that you [the GP] could hear. (FG H, RN1)

Although some RNs framed these added tasks as being manageable with less critically ill patients, they were worried that more complex cases might be missed or incorrectly assessed without a GP present. The RNs’ talk produced their dual responsibility to the patient and the GP as a state of uncertainty in which they had to rely on their own judgment without sufficient training, while managing an increased workload under the risk of missing critical cues. This allowed the RNs to manage their accountability by taking up a troubled position. As one newly examined RN said,

There is so much missing in nurses’ primary education, like how to examine the abdomen, lungs, heart, eyes, lymphatic system, everything. In a workplace like this, we must be able to do that. (FG Q RN2)

These examinations are constructed as necessary yet insufficiently supported by existing training. GPs, on the other hand, oriented to concerns about their own inability to perform hands-on clinical assessments, including concerns about ‘piecing together a diagnosis’ without vital clinical details and sensory information, which they presented as crucial for patient assessment. For example, the GPs referred to their own limitations in the distributed setting, such as their inability to observe the patient’s skin colour or use their own senses of sight, hearing and smell to collect information. By doing so, they positioned themselves as clinicians constrained by the conditions of the distributed setting, allowing them to account for diagnostic uncertainty and justify reliance on the RNs’ assessments. One GP made sense of this limitation:

I was trained in the 90s, when mobile phones were scarce, so I’m drilled in clinical assessment and medical history. Here, you can get a medical history through the nurse, but you don’t get a clinical assessment. (FG H, GP)

Other GPs framed this loss of hands-on assessments as losing ‘tools in the GP’s toolbox’ that were essential for making accurate diagnoses. By describing themselves as having to rely on RNs’ assessments rather than their own – particularly when working with RNs they were unfamiliar with – the GPs positioned themselves as dependent on others’ accounts, which enabled them to deflect responsibility in the distributed setting.

Similarly, the on-site nurses referred to their unfamiliarity with the GP on the screen as a way to justify uncertainty in decision-making and position themselves as working under constrained conditions. trust.

Taken together, these accounts construct a situation in which responsibility for clinical assessment is redistributed across distance, while access to clinical information remains uneven. RNs position themselves as required to perform and convey assessments that extend beyond their formally claimed competence, while GPs position themselves as dependent on information they cannot directly verify. Both positions construct a dilemma in which action becomes necessary yet remains uncertain, leaving the speakers potentially accountable as they make clinical decisions without full access to either competence or context.

### Control and dependency

Participants drew on the interpretative repertoire of *control and dependency* when they framed the distributed setup as introducing vulnerabilities related to both patient safety and professional responsibility. Both the RNs and the GPs oriented to technological dependence, organisational arrangements and the redistribution of responsibility across distance as sources of risk.

More specifically, the GPs oriented to concerns about losing control over the immediate patient care environment, managing uncertainties regarding their responsibilities, and working within a system that relies heavily on technology. Some GPs positioned themselves as having reduced control and influence, constructing organisational arrangements as having ‘forced’ them into this way of working, thereby placing them in a troubled position. These changes, presented as being driven by political and economic priorities, led the GPs to question their ability to take full responsibility for patient outcomes. They positioned themselves as increasingly dependent on a technology that remained outside their control. As one GP put it,

One must understand that this [telemedicine] can work as a complement, but I don’t think you can build a system around the idea that this will always work, because then you risk lives, in my opinion – a complement, nothing more. We need to be cautious about making organisational changes that remove on-call services because, suddenly, you are entirely dependent on the technology working, which it will not. It doesn’t matter what the tech guys say – it will never work every day, every hour, every second. It will fail. (FG H, GP)

Here, technological dependence is framed as a potential threat to patient safety, and the GP is positioned as accountable within a system the GP does not fully control. While the RNs also oriented to concerns about technological dependence, their accounts extended to the on-site situation, where they positioned themselves as the first point of contact for patients without immediate access to the GP. The RNs framed themselves as isolated and carrying responsibility during critical situations, managing patient care alongside NAs while waiting to establish contact with the GP via videoconferencing, often without sufficient knowledge of the system or access to technical support. They described themselves as being called to make decisions under conditions of uncertainty and delayed support, thereby positioning themselves as troubled. As one RN explained,

We will feel lonely and abandoned because of where we live and the logistics; the ambulance can be 1.5 h away if things go badly. Now that we share the on-call doctor with all the other locations [the other CHs], you don’t know in what order they will prioritise. If we have a critically ill patient and we call the doctor but they’re busy, sure, we can call 112 [Sweden’s emergency service number], but how do we know when we can reach the doctor? (FG J, RN1)

In this account, the RN frames uncertainty in relation to access, timing and availability, where responsibility for immediate action remains with the RN despite limited control over when support can be obtained, thereby accounting for the challenges of decision-making under constrained conditions.

The participants constructed the distributed setting as shaping communication in ways that could be both constraining and enabling. They presented communication as becoming more focused and deliberate through technology, by describing the team members as needing to listen carefully and avoid overlaps in interaction. They also talked of communication as requiring greater discipline and coordination in time-critical situations. As one RN said,

Communication becomes clearer online because grasping things is vital for all parties. Then, there’s more willingness and ability to stay silent longer and make an effort. It’s easy to interrupt each other when things heat up, and everyone is in the same place. (FG A, RN1)

Here, the RN frames communication as being more structured and controlled in a distributed setting, enabling clearer exchanges in acute situations. However, the RN also orients to tension within communication, as the communication between team members becomes dependent on technological mediation and timing in a distributed setting, making it more vulnerable to disruption.

In summary, the participants drawing on this repertoire construct a dilemma in which they maintain responsibility, while experiencing reduced control over the conditions for fulfilling that responsibility. GPs position themselves as accountable despite lacking direct access to the patient, while RNs position themselves as being required to act under conditions of uncertainty and limited support. In this way, both groups of participants place themselves in troubled positions. At the same time, they construct communication as being more structured and controlled in a distributed setting, suggesting an untroubled position that remains dependent on stable technological conditions.

## Discussion

In this study, participants accounted for acting under conditions where responsibility was not readily transferred, involvement was unevenly sustained, and control depended on others’ accounts in distributed emergency care. These dimensions reflect core components of teamwork described in healthcare and team science literature, such as communication, coordination and shared responsibility [19, 20], but in the present study, these aspects were constructed differently in the distributed setting compared to co-located teamwork.

In situations where competing and conflicting types of knowledge are involved, Billig [25] describes so-called ideological dilemmas. In this study, such dilemmas became apparent in the distributed setting, where established ways of organising teamwork, such as roles, responsibility and presence, are challenged.

Across the repertoires, responsibility was not simply transferred between professionals but had to be managed under different constraints, as on-site staff assumed immediate responsibility for patient care while remote GPs relied on second-hand accounts. RNs constructed themselves as taking on tasks beyond their usual scope, balancing increased autonomy with concerns about their clinical competence. Rather than simply reflecting a redistribution of tasks, the RNs framed this shift as placing them at the forefront of patient care and their assessments as critical in guiding the GPs’ decisions.

This finding aligns with research on task-shifting, in which responsibilities are redistributed across professional boundaries [44, 45], often in response to resource constraints. However, as highlighted by the World Health Organisation, expanding roles without adequate preparation may increase stress and risk, particularly in high-pressure environments [44]. In our findings, this was evident in how the RNs oriented to gaps in training and uncertainty in more complex cases. What stands out here is not only that tasks shift, but that accountability does not shift as easily, leaving RNs to act while managing both expectations of competence and potential consequences of error.

The GPs, in turn, positioned themselves within a dilemma of control and dependence. While they framed the distance as allowing them to take on a more observational and less stressful role, they simultaneously presented it as limiting their access to the clinical situation. The GPs managed their accountability in the distributed setting by emphasising that their ability to make decisions relied on information produced by others and mediated through technology, rather than on direct assessment.

Previous research has shown that distributed teamwork often involves challenges related to trust and familiarity [8, 46], particularly when team members are required to act on information they cannot verify themselves. In our study, this was further complicated by the ad-hoc nature of rural teams, where opportunities to build familiarity are limited [16]. Teams that only meet sporadically and therefore lack a deeper understanding of each other’s skills can struggle to establish trust [8, 47]. This difficulty is worsened by digital interactions, which make trust-building even harder. Although scheduling ‘get-to-know-you’ meetings beforehand may solve this problem [16], doing so may be unrealistic in rural healthcare settings. As a result, the participants constructed control as being maintained in principle but dependent on others’ assessments and the reliability of communication and technology in practice.

The dilemma of involvement was closely tied to how participation was made visible and sustained in interaction. While the participants constructed distributed teamwork as enabling involvement and support, the findings showed that involvement was fragile and unevenly distributed. Being involved was a matter not only of being connected through technology but of being acknowledged within the interaction. By describing difficulties in maintaining shared attention, interpreting non-verbal cues and coordinating communication across distance, different team members positioned themselves as peripheral at different moments. This finding aligns with previous research showing that distributed communication places higher demands on coordination and increases the risk of misalignment [13].

These qualitative findings can be considered in relation to a simulation-based observational study conducted within the same research programme and described in the methods [27], in which co-located teams showed higher overall performance, particularly in completing tasks on time and adapting to changing situations. Rather than interpreting this as a simple performance deficit, the present findings suggest that these challenges emerge from how teamwork is organised across distance, where coordination, timing and decision-making depend on mediated communication and distributed responsibility.

The result of this study further suggests that distributed teamwork is not simply a variation of co-located teamwork but requires specific competencies and targeted training to function safely across distance. From a broader interprofessional perspective, these findings point to a reconfiguration of roles and authority within the team. Traditional medical discourse positions physicians as central decision-makers who control information flow and lead the team [48–50]. In the distributed setting, this position becomes less stable, as decision-making depends on contributions from those physically present with the patient.

At the same time, increased responsibility for RNs does not necessarily translate into increased authority, but rather into a more exposed position where they must act while relying on remote support. This creates a persistent tension between responsibility and control, in which acting and deciding are no longer in the same place.

Finally, despite our encouragement, the NAs had little vocal participation in the focus groups. However, they still played a crucial role in the team, particularly in a distributed setup. Their physical presence with the patient gave the NAs direct access to observe and manage patient conditions. Regardless of their central role in care, due to their team functions and level of training, the NAs often remained on the sidelines in decision-making. This duality, which is central to patient care yet marginalised in team discussions, reflects the hierarchical dynamics within the team. The NAs’ lack of participation in the discussions did not mean they contributed less to the team; rather, it highlighted how institutional structures and hierarchies influence who gets to speak and when [49, 51].

### Limitations

The time gap between data collection (2019–2021) and analysis is a limitation, as developments in digitalisation may have influenced the context of distributed teamwork during this gap. In addition, half of the data collection occurred during the COVID-19 pandemic, a period of considerable healthcare pressure that may have shaped participants’ accounts, particularly in relation to workload, uncertainty and the use of digital solutions. However, as distributed models of care and remote collaboration have continued to develop, the findings remain relevant for understanding how teamwork is organised and negotiated across distance. Another limitation is that using a sample from a small geographical area may limit the generalisability of this study’s findings to other contexts or regions. However, the study’s interdisciplinary focus groups provided diverse perspectives on distributed teamwork. The simulated team training conducted before the interviews added realism to the discussions and may have increased the validity of the findings. Furthermore, variations in the physical setup of the videoconferencing systems used across the seven CHs in southern Lapland may have influenced how the participants narrated their experiences and perceived the teamwork among the groups. Screen placement varied between rooms, with some positioned behind or in front of the patient and others mounted overhead. To enhance the credibility and authenticity of the findings, we have included excerpts from the interviews, which help verify the accuracy of our interpretations and ensure transparency in the presentation of the data.

### Reflexivity

Reflecting on our assumptions and biases as healthcare professionals and researchers, we recognise that these factors may have shaped our approach to the study and interpretation of the findings. We sought to self-monitor and remain aware of our pre-understandings throughout the research process.

At the time of the interviews, HM was a female PhD candidate and midwife, HD was a female PhD candidate and RN (emergency), and MHä was a female PhD and RN (anaesthesia). MHu and JC were male MDs and PhDs in anaesthesiology. All five authors were trained simulation instructors and had professional experience in emergency care. None of the researchers had prior experience working in rural settings or in distributed teams using telemedicine.

As interviewers (MHä, HD and HM), we attempted to balance participation among the different professions in the focus groups. Nevertheless, we noticed a power dynamic in which the NAs spoke the least. As all interviewers were RNs, it is possible that we were unconsciously part of this hierarchy. Furthermore, none of the researchers had a prior working relationship with the participants, although one interviewer (MHä) grew up in the region.

Our shared familiarity with high-stress clinical settings may have shaped how the participants’ experiences were understood and interpreted. In some cases, we may have recognised or related to the challenges and dilemmas described, which could have increased our sensitivity to certain aspects of the data while creating a risk of blind spots in others. To confirm the trustworthiness of the research process, we held continuous discussions among the authors, bringing together diverse medical and methodological perspectives. The mix of nursing and physician backgrounds, along with different levels of research experience, supported critical reflection and helped challenge assumptions throughout the analytical process.

## Conclusion

Our findings suggest that distributed teamwork reshapes the conditions for collaboration in emergency care. Rather than reflecting stable roles and clearly defined responsibilities, teamwork becomes dynamic and continuously negotiated, as participants position themselves and others in relation to tensions concerning involvement, responsibility and control. These ways of accounting for action show how team members come to occupy different positions across situations, and that effective collaboration cannot be taken for granted in distributed settings.

## Data Availability

No datasets were generated or analysed during the current study.

## Abbreviations

CH: Community hospital
COREQ: Consolidated Criteria for Reporting Qualitative Results
DP: Discourse psychology
GP: General practitioner
NA: Nursing assistant
RN: Registered nurse
TIGER: Teamwork in Geographically Dispersed Emergency Teams in Rural Settings

## References

[1] Kohn L, Corrigan JMD, Donaldson MS. To err is human: building a safer health system. Washington, DC: National Academy Press; 2000. ISBN-10: 0-309-06837-1. 25077248

[2] Hjortdahl M, Ringen AH, Naess AC, Wisborg T. Leadership is the essential non-technical skill in the trauma team - results of a qualitative study. Scand J Trauma Resusc Emerg. 2009;17:48. 10.1186/1757-7241-17-48 PMC2764560 19781093

[3] Härgestam M, Hultin M, Brulin C, Jacobsson M. Trauma team leaders’ non-verbal communication: video registration during trauma team training. Scand J Trauma Resusc Emerg. 2016;24(1). 10.1186/s13049-016-0230-7 PMC4807541 27015914

[4] Manser T. Teamwork and patient safety in dynamic domains of healthcare: a review of the literature. Acta Anaesthesiol Scand. 2009;53:143–51. 19032571 10.1111/j.1399-6576.2008.01717.x

[5] Hughes AM, Sonesh SC, Mason RE, Gregory ME, Marttos A, Schulman CI, et al. Trauma, teams, and telemedicine: evaluating telemedicine and teamwork in a mass casualty simulation. Mil Med. 2021;186. 10.1093/milmed/usaa434 33216935

[6] Al-Ani B, Horspool A, Bligh MC. Collaborating with ‘virtual strangers’: towards developing a framework for leadership in distributed teams. Leadersh Q. 2011;7(3):219–49.

[7] Fiore SM, Salas E, Cuevas HM, Bowers CA. Distributed coordination space: toward a theory of distributed team process and performance. Theor Issues Ergon Sc. 2003;4(3):340–64.

[8] Anderson AH, McEwan R, Bal J, Carletta J. Virtual team meetings: an analysis of communication and context. Comput Hum Behav. 2007;23:2558–80.

[9] Connaughton S, Shuffler M, Goodwin GF. Leading distributed teams: the communicative constitution of leadership. Mil Psychol. 2011;23(5):502–27.

[10] Avolio BJ, Sosik JJ, Kahai SS, Baker B. E-leadership: re-examining transformations in leadership source and transmission. Leadersh Q. 2014;25:105–31.

[11] White B, Johnson J, Arroliga A, Couchman G. Ad hoc teams and telemedicine during COVID-19. Proc (Bayl Univ Med Cent). 2020;33:696–8. 33100575 10.1080/08998280.2020.1809758 PMC7549959

[12] Miloslavic SA, Wildman JL, Thayer AL. Structuring successful global virtual teams. In: Wildman JL, Griffith RL, editors. Leading global teams: translating multidisciplinary science to practice. New York: Springer Science; 2015. pp. 67–87.

[13] Gibbs JL, Sivunen A, Boyraz M. Investigating the impacts of team type and design on virtual team processes. Hum Resour Manag Rev. 2017;27:590–603.

[14] Avolio BJ, Kahai S, Dumdum R, Sivasubramaniam N. Virtual teams: implications for e-leadership and team development. In: London E, editor. How people evaluate others in organizations. Mahwah, NJ: Lawrence Erlbaum Associates; 2001. pp. 337–58.

[15] Butler L, Whitfill T, Wong AH, Gawel M, Crispino L, Auerbach M. The impact of telemedicine on teamwork and workload in pediatric resuscitation: a simulation-based, randomized controlled study. Telemed J e-Health. 2019;25:205–12. 29957150 10.1089/tmj.2018.0017

[16] Saunders CS, Ahuja MK. Are all distributed teams the same? Differentiating between temporary and ongoing distributed teams. Small Group Res. 2006;37:662–700.

[17] Stanton NA, Plant KL, Revell KMA, Griffin TGC, Moffat S, Stanton M. Distributed cognition in aviation operations: a gate-to-gate study with implications for distributed crewing. Ergon. 2019;62:138–55. 10.1080/00140139.2018.1520917 30192716

[18] Hoch JE, Kozlowski SW. Leading virtual teams: hierarchical leadership, structural supports, and shared team leadership. J Appl Psychol. 2014;99:390–403. 23205494 10.1037/a0030264

[19] Salas E, Sims DE, Burke CS. Is there a Big Five in teamwork? Small Gr Res. 2005;36(5):555–99.

[20] Rosen MA, DiazGranados D, Dietz AS, Benishek LE, Thompson D, Pronovost PJ, Weaver SJ. Teamwork in healthcare: Key discoveries enabling safer, high-quality care. Am Psychol. 2018;73(4):433–50. 29792459 10.1037/amp0000298 PMC6361117

[21] Berggren P, Pourazar E, Lundqvist A, Sweden. Access to rural services by strengthening primary care with digital tools in remote areas of Sweden. Geneva: WHO; 2022.

[22] Hedman M. The community hospital model in northern Sweden. Umeå: CityPrint i Norr AB: Umeå University; 2024.

[23] Wetherell M, Taylor S, Yates SJ. Discourse as data: a guide for analysis. London: Sage; 2001.

[24] Wetherell M. Positioning and interpretative repertoires: conversation analysis and post-structuralism in dialogue. Discourse Soc. 1998;9:387–412.

[25] Billig M, Condor S, Edwards DMG, Middleton DAR. Ideological dilemmas: a social psychology of everyday thinking. London: Sage; 1988.

[26] Morian H, Creutzfeldt J, Hultin M, Härgestam M. Mapping leadership, communication and collaboration in short-term distributed teams across various contexts: a scoping review. BMJ Open. 2024;14:e081878. 39448210 10.1136/bmjopen-2023-081878 PMC11499798

[27] Morian H, Hultin M, Lindkvist M, Creutzfeldt J, Dubois H, Jonsson K, et al. Teamwork in rural emergency health care: a simulation-based cross-over study of co-located and distributed teams. Simul Healthc. 2024. 10.1097/SIH.0000000000000831 PMC12129384 39417732

[28] Morian H, Härgestam M, Hultin M, Jonsson H, Jonsson K, Nordahl Amorøe T, et al. Reliability and validity testing of team emergency assessment measure in a distributed team context. Front Psychol. 2023;14:1110306. 37151315 10.3389/fpsyg.2023.1110306 PMC10157038

[29] Dubois H, Bergenmar M, Härgestam M, Creutzfeldt J. Patient participation in tele-emergencies - experiences from healthcare professionals in northern rural Sweden. Rural Remote Health. 2022;22:7404. 36480908 10.22605/RRH7404

[30] Ärlebrant L, Dubois H, Creutzfeldt J, Edin-Liljegren A. Emergency care via video consultation: interviews on patient experiences from rural community hospitals in northern Sweden. Int J Emerg Med. 2024;17:109. 39227787 10.1186/s12245-024-00703-4 PMC11370045

[31] Dubois H, Creutzfeldt J. Behavioural observation tool for patient involvement and collaboration in emergency care teams (PIC-ET-tool). BMC Emerg Med. 2023;23:74. 37393240 10.1186/s12873-023-00841-7 PMC10314478

[32] Dubois H, Manser T, Häbel H, Härgestam MJC. Exploring differences in patient participation in simulated emergency cases in co-located and distributed rural emergency teams –an observational study with a randomized cross-over design. BMC Emerg Med. 2024;24:18. 39009973 10.1186/s12873-024-01037-3 PMC11247836

[33] Tong A, Sainsbury P, Craig J. Consolidated Criteria for Reporting Qualitative Research (COREQ): a 32-item checklist for interviews and focus groups. Int J Qual Health Care. 2007;19:349–57. 17872937 10.1093/intqhc/mzm042

[34] Hedman M, Boman K, Brännström M, Wennberg P. Clinical profile of rural community hospital inpatients in Sweden - a register study. Scand J Prim Health Care. 2021;39:92–100. 33569976 10.1080/02813432.2021.1882086 PMC7971215

[35] World Health Organisation. World health statistics 2016: monitoring health for the SDGs, sustainable development goals. Geneva: WHO. 2016. ISBN: 9789241565264.

[36] Swedish National Agency for Education (Skolverket). Health and social care program: specializations and courses. 2020. https://www.skolverket.se2020. Accessed 10 Oct 2024.

[37] Swedish Society of Nursing (Svensk Sjuksköterskeförening). Competency description for registered nurses. 2024. ISBN: 978-91-85060-74-0.

[38] Swedish Medical Association (Läkarförbundet). Medical education and specialist training: an overview. 2020. https://www.slf.se. Accessed 10 Oct 2024.

[39] Helmreich RL, Merritt AC, Wilhelm JA. The evolution of Crew Resource Management training in commercial aviation. Int J Aviat Psychol. 1999;9(1):19–32. 11541445 10.1207/s15327108ijap0901_2

[40] Crawford SB, Baily LW, Monks SM. Comprehensive Healthcare Simulation: Operations, Technology, and Innovative Practice. Switzerland: Springer; 2019.

[41] Krueger RA. Focus groups: a practical guide for applied research. Sage; 2014.

[42] Potter J, Wetherell M. Discourse and social psychology: beyond attitudes and behaviour. London: Sage; 1987.

[43] Dubois A, Gadde L-E. Systematic combining: an abductive approach to case research. J Bus Res. 2002;55:553–60.

[44] World Health Organisation. Task shifting: rational redistribution of tasks among health workforce teams: global recommendations and guidelines. World Health Organ. 2008. ISBN 978 92 4 159631 2.

[45] Frenk J, Chen L, Bhutta ZA, Cohen J, Crisp N, Evans T, et al. Health professionals for a new century: transforming education to strengthen health systems in an interdependent world. Lancet. 2010;376(9756):1923–58. 21112623 10.1016/S0140-6736(10)61854-5

[46] Espinosa JA, Slaughter SA, Kraut RE, Herbsleb JD. Familiarity, complexity, and team performance in geographically distributed software development. Organ Sci. 2007;18:613–30.

[47] Rockwood J, Nathan-Roberts D. A systematic review of communication in distributed crews in high-risk environments. Proc Hum Factors Ergon Soc. 2018;102–6.

[48] Hershkovich O, Gilad D, Zimlichman E, Kreiss Y. Effective medical leadership in times of emergency: a perspective. Disaster Mil Med. 2016;2. 10.1186/s40696-016-0013-8 PMC5329946 28265438

[49] Reeves S, Lewin S, Espin S, Zwarenstein M. Interprofessional teamwork for health and social care: Partnership working in action. Wiley-Blackwell; 2010.

[50] World Health Organization. Framework for action on interprofessional education and collaborative practice. Geneva: World Health Organization; 2010. 21174039

[51] Foucault M. In: Gordon C, editor. Power/knowledge: selected interviews and other writings, 1972–1977. New York: Pantheon Books; 1980.

